# Deregulation of the miR-19b/PPP2R5E Signaling Axis Shows High Functional Impact in Colorectal Cancer Cells

**DOI:** 10.3390/ijms24097779

**Published:** 2023-04-24

**Authors:** Andrea Santos, Ion Cristóbal, Cristina Caramés, Melani Luque, Marta Sanz-Álvarez, Juan Madoz-Gúrpide, Federico Rojo, Jesús García-Foncillas

**Affiliations:** 1 Cancer Unit for Research on Novel Therapeutic Targets, Oncohealth Institute, IIS-Fundación Jiménez Díaz-UAM, 28040 Madrid, Spain; 2 Translational Oncology Division, Oncohealth Institute, IIS-Fundación Jiménez Díaz-UAM, 28040 Madrid, Spain; 3 Medical Oncology Department, University Hospital “Fundación Jiménez Díaz”, UAM, 28040 Madrid, Spain; 4 Pathology Department, IIS-Fundación Jiménez Díaz-UAM, 28040 Madrid, Spain

**Keywords:** miR-19b, PPP2R5E, CRC

## Abstract

MicroRNA (miR)-19b is deregulated in colorectal cancer (CRC) and locally advanced rectal cancer (LARC), predicting worse outcome and disease progression in CRC patients, and acting as a promising prognostic marker of patient recurrence and pathological response to 5-fluorouracil (5-FU)-based neoadjuvant chemoradiotherapy in LARC. Moreover, there is a strong inverse correlation between miR-19b and PPP2R5E in LARC, and both predict the response to neoadjuvant therapy in LARC patients. However, the functional role of the miR-19b/PPP2R5E axis in CRC cells remains to be experimentally evaluated. Here, we confirm with luciferase assays that miR-19b is a direct negative regulator of PPP2R5E in CRC, which is concordant with the observed decreased PP2A activity levels after miR-19b overexpression. Furthermore, PPP2R5E downregulation plays a key role mediating miR-19b-induced oncogenic effects, increasing cell viability, colonosphere formation ability, and the migration of CRC cells. Lastly, we also confirm the role of miR-19b mediating 5-FU sensitivity of CRC cells through negative PPP2R5E regulation. Altogether, our findings demonstrate the functional relevance of the miR-19b/PPP2R5E signaling pathway in disease progression, and its potential therapeutic value determining the 5-FU response of CRC cells.

## 1. Introduction

Colorectal cancer (CRC) constitutes one of the most globally prevalent cancers as the second most common cause of cancer death, and third in terms of cancer incidence, comprising 11% of all cancer diagnoses [1,2,3]. About one-third of CRCs are identified as rectal cancer (RC), which, in 2020, corresponded to approximately 732,210 new cases globally [4,5]. Patients diagnosed with early RC can usually proceed with the direct surgical removal of the tumor, showing good outcome and a low incidence of recurrences. However, a significant number of patients have a locally advanced disease and are optimal candidates to benefit from an alternative strategy that aims to downstage the tumor prior to surgery [6]. Locally advanced rectal cancer (LARC) is mostly defined as clinical stage T3–4 or any clinical T stage with lymph-node-positive disease (≥cT_3–4_ or any cT with cN_1/2_) whose management is particularly challenging due to the anatomical characteristics of the pelvis together with the higher local recurrence risk [7,8]. Fluorouracil-based preoperative chemoradiotherapy represents the standard neoadjuvant treatment of LARC patients, followed by a total mesorectal excision (TME) and postoperative adjuvant chemotherapy [9,10]. Although this therapeutic approach has progressively achieved both reduced local recurrence rates and improved patient surveillance, disease progression still affects a large number of cases, representing a subgroup of patients with worse outcome [9]. Therefore, it is necessary to better understand the molecular mechanisms that govern RC progression in order to identify and incorporate novel biological predictive markers in the clinical practice that would allow for us to anticipate and prevent tumor progression in these patients.

In the last decade, an increasing number of studies have identified microRNAs (miRs) as useful predictive biomarkers for cancer diagnosis, prognosis, and therapeutic response. miRs also play essential roles as oncogenes or tumor suppressors, depending on their regulated targets, but the molecular mechanisms by which they function mostly need to be fully investigated and validated [11,12]. This class of small noncoding RNA molecules bind the 3′untranslated region of their target messenger RNAs (mRNAs), regulating their expression by repressing mRNA translation and triggering its degradation [11,12,13,14]. As indicated above, miRs act as regulators of a variety of cellular processes, including proliferation, cell cycle, apoptosis, differentiation, and stress response [12,14,15]. Thus, miRs have are highly deregulated in cancer cells, inducing the development and progression of many cancer types, such as triple-negative breast cancer, hepatocellular and prostate cancer, colorectal cancer, and nonsmall cell lung cancer (NSCLC) [16,17,18,19].

MiR-19b is a major oncogenic miR of the miR-17-92 cluster that plays a central role in tumor progression [20]. Thus, high miR-19b expression leads to increased ERK, AKT, and STAT phosphorylation and activation in NSCLC, resulting in enhanced cell-cycle progression, cell viability and migration, and reduced apoptosis [21]. The deregulation of tumor suppressor PTEN, a direct target of miR-19b, is involved in miR-19b-induced epithelial–mesenchymal transition in lung cancer and multiple myeloma [21,22,23]. In addition, miR-19b overexpression reduces p53 protein levels, which results in the downregulation of proapoptotic proteins such as Bax and p21, and the consequent enhancement of tumor growth and metastasis [24]. However, limited experimental research has been performed to analyze the molecular mechanisms underlying the miR-19b contribution to CRC progression. The upregulation of miR19b-3p/ITGB8 axis plays an important role in the growth and metastasis of CRC cells [25]. Moreover, the work of Jiang et al. confirmed that this miR mediates resistance to oxaliplatin-based chemotherapy through SMAD4 regulation, promoting colon cancer proliferation [26] and mediating cell-cycle progression [27]. In addition, miR-19b predicts shorter overall survival and metastatic progression in CRC patients [28]. In concordance, previous studies from our group showed that miR-19b is highly deregulated in LARC, and predicts both patient outcome and pathological response to neoadjuvant chemoradiotherapy (nCRT), and cancer progression in this disease, observing higher recurrence rates in patients with high miR-19b levels [29,30]. Moreover, PP2A regulatory subunit PPP2R5E was able to predict the response to nCRT in our LARC patient cohort, confirming the inverse correlation that exists between miR-19b and PPP2R5E expression in this disease [29]. In fact, PPP2R5E is a direct miR-19b target that plays a relevant role in NSCLC progression, mediating the miR-19b-induced PP2A inhibition in this disease [21]. Moreover, Mavrakis et al. identified PPP2R5E as one of the knockdown genes that are between the miR-19b targets responsible for its oncogenic action in acute lymphoblastic leukemia [31].

We previously identified PPP2R5E downregulation as a molecular contributing mechanism to inactivate PP2A in CRC [32], and PP2A inhibition as an alteration responsible to decrease 5-FU antitumor effects [33]. These findings prompted us to evaluate the role of miR-19b as a modulator of 5-FU response, showing that this miR plays a key role in the 5-FU-resistant phenotype of CRC cells. However, the potential contribution of PPP2R5E to these effects through the miR-19b/PPP2R5E axis needs to be clarified. Moreover, it remains necessary to explore the functional role of miR-19b/PPP2R5E, which would strengthen the reported clinical value of this signaling axis in both CRC and LARC, and validate these actors as novel molecular targets in these diseases.

## 2. Results

### 2.1. MiR-19b Plays a Relevant Role in Regulating the Cell Viability of CRC Cells

**Figure 1:**
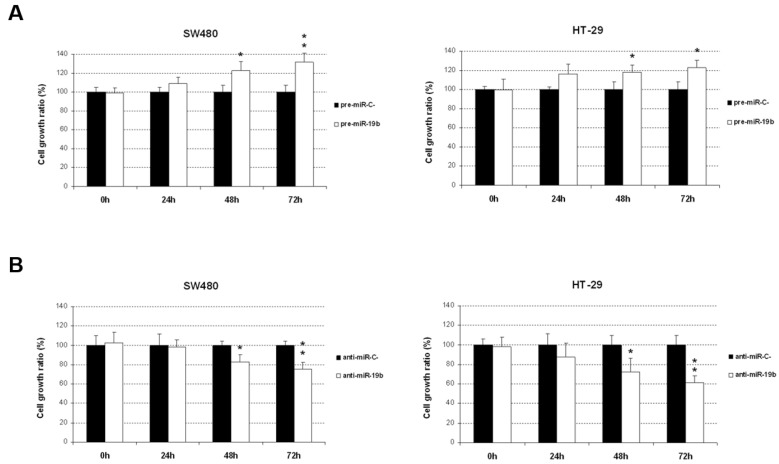
Ectopic modulation of miR-19b induces changes in CRC cell viability. MTS assays were performed in SW480 and HT-29 cells after specific miR-19b (**A**) overexpression or (**B**) silencing; * *p* < 0.05; ** *p* < 0.01.

To assess the biological relevance of miR-19b deregulation in tumor progression, we first evaluated the potential effects of an ectopic modulation of this miR in cell viability. SW480 and HT-29 cells transfected with pre-miR-19b showed significantly increased cell viability compared to that of the negative controls (Figure 1A). A remarkable reduction in cell viability, on the other hand, was found after miR-19b inhibition (Figure 1B).

Given that PPP2R5E is a direct target of miR-19b, we also analyzed the role of PPP2R5E in cell viability, observing that PPP2R5E overexpression led to decreased proliferation in both cell lines in comparison with cells transfected with an empty vector (Figure S1), and the opposite effect after PPP2R5E silencing (Figure S2). Altogether, these findings indicate that miR-19b and PPP2R5E modulation plays an important role regulating CRC cell viability.

### 2.2. Significant Relevance of miR-19b-Mediated PP2A Activity Inhibition through the Direct Negative Regulation of PPP2R5E

**Figure 2:**
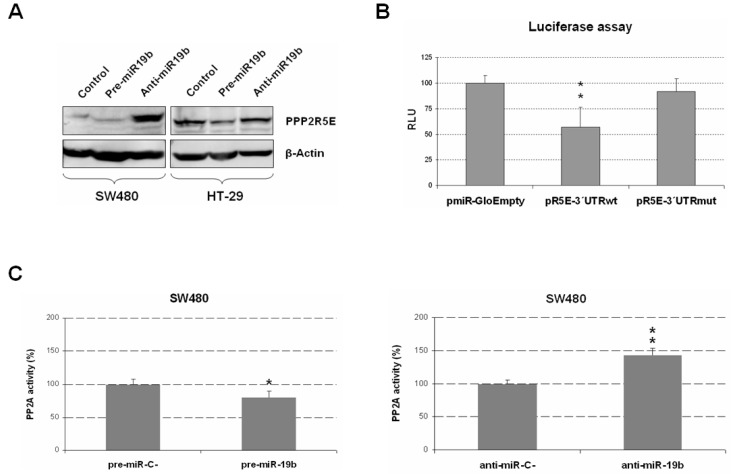
Functional role of miR-19b regulating PPP2R5E expression and PP2A activity. (**A**) Analysis of PPP2R5E protein expression changes via Western blot after ectopic miR-19b modulation. (**B**) Evaluation via luciferase assays of the role of miR-19b as a direct negative regulator of PPP2R5E. Normalized luciferase activity (firefly luciferase activity/Renilla luciferase activity) for each construct was compared to that of the pmiR-Glo vector with no insert as the control. (**C**) PP2A phosphatase activity assays in SW480 cells after miR-19b overexpression or silencing. Graphs show the mean of three independent experiments ± SD; * *p* < 0.05; ** *p* < 0.01.

Using a Western blot, we next analyzed the effect of miR-19b modulation on PPP2R5E expression in SW480 and HT-29 cell lines overexpressing or silencing miR-19b with specific pre- and anti-miRs for miR-19b. As expected, miR-19b overexpression led to a reduction in PPP2R5E protein levels, especially in HT-29 cells, since the basal PPP2R5E levels were higher than those in the SW480 cell line. In concordance with these results, we found increased PPP2R5E expression after transfection with anti-miR-19b, which was particularly evident in SW480 cells in which the control cells showed lower levels of this protein than those of HT-29 cells (Figure 2A).

To test whether miR-19b was a post-transcriptional regulator of PPP2R5E in our tumor cells, we performed a luciferase assay. Interestingly, we observed a significant decrease in luciferase activity in SW480 ectopically expressing miR-19b that had been transfected with a plasmid vector, including the 3′UTR region of PPP2R5E with the predicted seed region for this miR. However, the same experiment performed on the mutated miR-19b seed region did not show changes in luciferase activity, indicating that miR-19b directly binds and regulates *PPP2R5E* mRNA (Figure 2B). Therefore, our findings validate that miR-19b is a direct negative regulator of PPP2R5E in CRC cells.

PPP2R5E is a PP2A regulatory subunit whose deregulation represents a contributing molecular alteration to PP2A inhibition in CRC cells. This issue prompted us to explore whether changes in miR-19b, as a negative regulator of PPP2R5E, could affect PP2A phosphatase activity. Thus, we performed a specific PP2A phosphatase activity assay after ectopic miR-19b modulation, and miR-19b overexpression led to significant PP2A inhibition, whereas miR-19b silencing enhanced PP2A activity levels in SW480 cells (Figure 2C). We further confirmed these observations by establishing PP2A phosphatase activity in HT-29 cells after ectopic miR-19b modulation that showed similar results (Figure S3).

### 2.3. MiR-19b Promotes Cell Proliferation in a PPP2R5E-Dependent Manner

**Figure 3:**
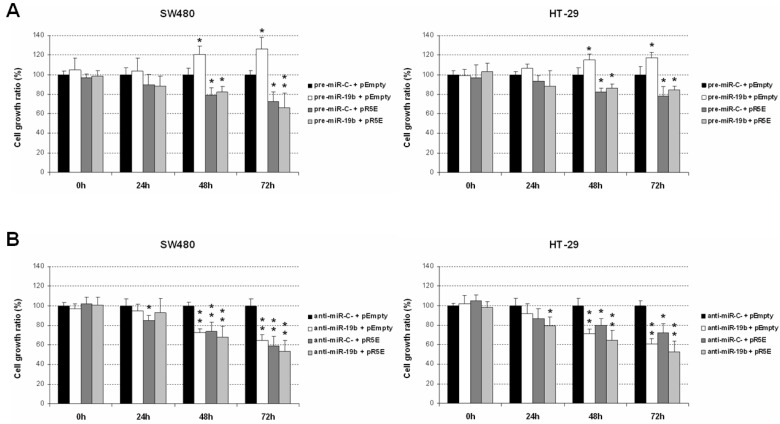
MiR-19b-induced effects on cell viability are mediated by its role as a direct PPP2R5E regulator. (**A**) Effect of PPP2R5E in miR-19b-overexpressing SW480 and HT-29 cells. (**B**) Effect of PPP2R5E in SW480 and HT-29 cells after miR-19-silencing. * *p* < 0.05; ** *p* < 0.01.

The demonstration of PPP2R5E as a direct miR-19b target prompted us to analyze the contribution of this protein to the functional properties regulated by miR-19b in CRC cells. Thus, we first investigated the role of the miR-19b/PPP2R5E signaling pathway in cell viability. PPP2R5E overexpression totally impaired the miR-19b-induced cell proliferation in both SW480 and HT-29 cells (Figure 3A). To further confirm these observations, we repeated these experiments with miR-19b silencing. In concordance with the above results, miR-19b inhibition with or without PPP2R5E overexpression led to marked decreased cell viability in both cell lines (Figure 3B). These findings confirmed that PPP2R5E regulation plays a key role mediating miR-19b-induced effects in cell viability, highlighting the relevance of the miR-19b/PPP2R5E axis modulating CRC cell viability.

### 2.4. MiR-19b Regulates Cell Migration and Induces Colonosphere Formation in CRC Cells

**Figure 4:**
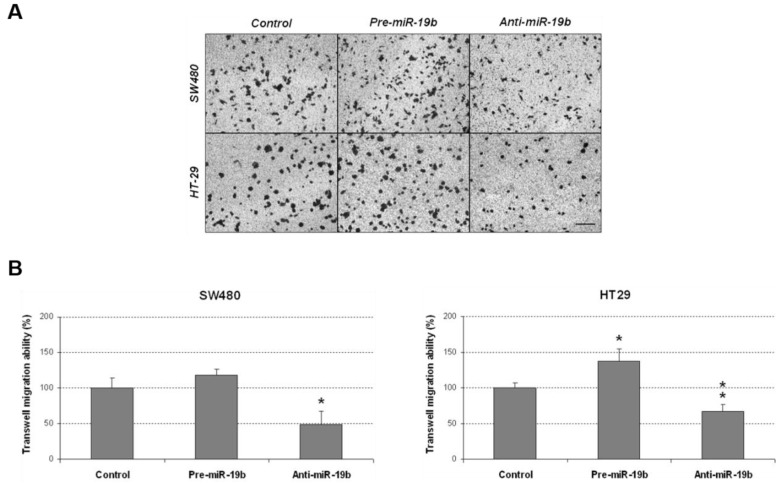
Transwell migration assay showing that miR-19b modulates the migration of CRC cells. (**A**) Optical microscopy images (amplification ×100) showing miR-19b-induced changes in cell migration. (**B**) Differences in migrated SW480 and HT-29 cells after ectopic miR-19b modulation; * *p* < 0.05; ** *p* < 0.01. Scale bar: 200 µm.

We analyzed additional miR-19b-induced oncogenic properties that could contribute to disease progression by evaluating changes in cell migration after its ectopic modulation. In concordance with its ability to regulate cell proliferation, we found that miR-19b silencing significantly reduced migration in SW480 cells, and similar results were observed in the HT-29 cell line. miR-19b overexpression induced the opposite effect, enhancing cell migration in both cases, but only achieving statistical significance in HT-29 cells (Figure 4).

We next analyzed the role of PPP2R5E in cell migration, performing transwell migration assays in SW480 and HT-29 cells, observing that PPP2R5E overexpression led to a marked reduction in cell migration ability in both cell lines (Figure S4). Migration was increased when PPP2R5E was silenced with a specific siRNA, even though significance was only obtained in HT-29 cells (Figure S5). We next confirmed the role of the miR-19b/PPP2R5E axis modulating miR-19b in SW480 and HT-29 cells ectopically expressing PPP2R5E (Figure S6), observing the PPP2R5E overexpression impaired miR-19b-induced migration. When we conducted the same experiments silencing PPP2R5E, miR-19b-induced migration was observed again (Figure S7). These results further confirm the role of the miR-19b/PPP2R5E axis as regulator of cell migration in CRC cells.

**Figure 5:**
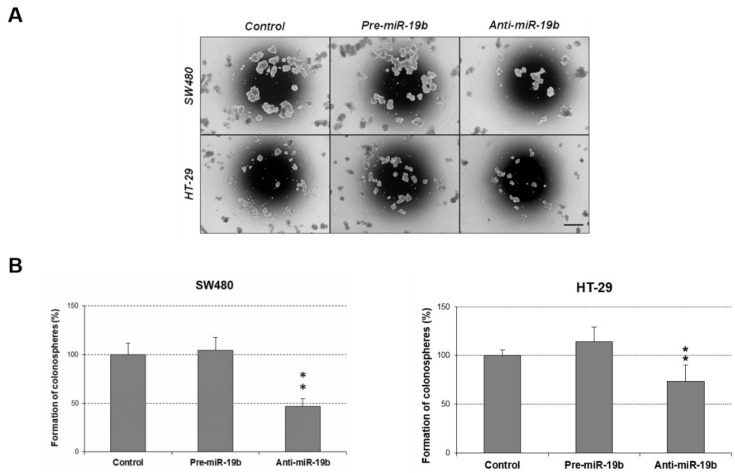
Role of miR-19b in the regulation of colonosphere formation. (**A**) Optical microscopy images (amplification ×100) showing colonospheres formed in different conditions. (**B**) Number of colonospheres obtained from SW480 and HT-29 cells after ectopic miR-19b modulation; ** *p* < 0.01. Scale bar: 200 µm.

We also evaluated the role of miR-19b in colonosphere formation ability of CRC cells. Remarkably, miR-19b downregulation led to a marked reduction in the number of colonospheres formed in both SW480 and HT-29 cells. miR-19b overexpression only caused a slight increment of the number of formed colonospheres that did not achieve statistical significance (Figure 5). However, miR-19b overexpression enhanced the colonosphere size in SW480 cells, and the opposite effect was observed when cells were transfected with anti-miR-19b (Figure S8).

We also analyzed the role of PPP2R5E regulating the colonosphere formation ability of CRC cells and found that its overexpression reduced the number of formed colonospheres by almost 50% (Figure S9). In addition, these results were confirmed by the fact that PPP2R5E downregulation led to an increased number of colonospheres in both SW480 and HT-29 cell lines (Figure S10). Lastly, we performed rescue experiments to validate the role of the miR-19b/PPP2R5E axis in colonosphere formation. Thus, we ectopically modulated miR-19b expression levels in SW480 and HT-29 cells after PPP2R5E silencing. PPP2R5E silencing totally impaired anti-miR-19b-induced effects in HT-29 cells, and markedly reduced those effects in the SW480 cell line (Figure S11), further supporting that miR-19b mainly regulates colonosphere formation through the miR-19b/PPP2R5E axis.

### 2.5. MiR-19b/PPP2R5E Axis Regulates 5-FU Resistance in CRC Cells

**Figure 6:**
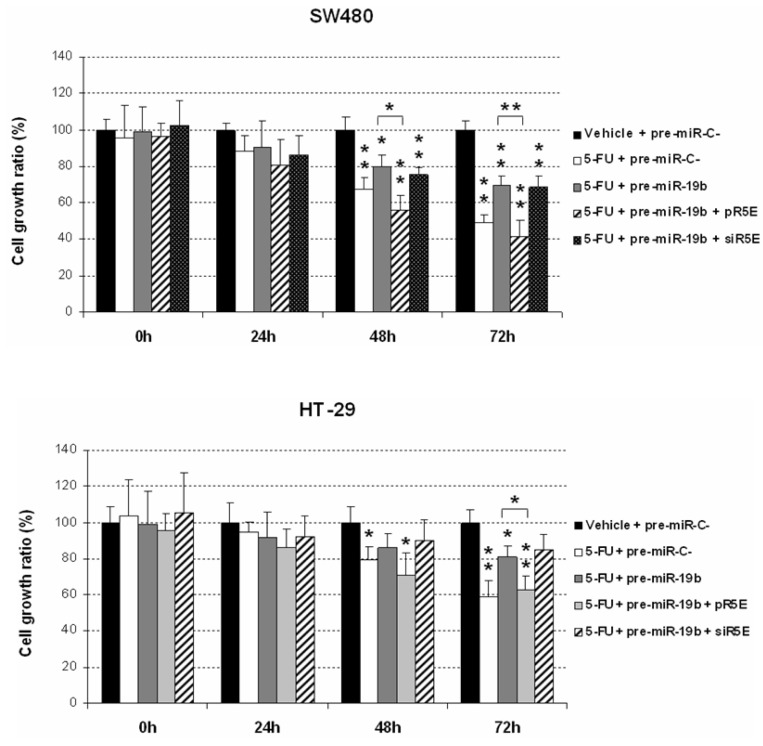
MTS assays showing the role of the miR-19b/PPP2R5E interplay in the regulation of CRC response to 5-FU treatment; * *p* < 0.05; ** *p* < 0.01.

Since miR-19b contributes to 5-FU resistance in CRC, we examined whether miR-19b affects 5-FU sensitivity through PPP2R5E inhibition. For this purpose, we modulated PPP2R5E expression in 5-FU treated CRC cells ectopically overexpressing miR-19b. As expected, miR-19b decreased 5-FU antitumor effects in SW480 cells. However, PPP2R5E overexpression completely restored 5-FU sensitivity of these cells, observing similar proliferation rates as in those cells only treated with the drug. Interestingly, the opposite effect was found after PPP2R5E silencing. These results were confirmed with the HT-29 cell line (Figure 6). Altogether, our findings highlight the relevance of the miR-19b/PPP2R5E signaling axis determining response of CRC cells to 5-FU treatment.

To further confirm these results, we performed apoptosis assays, observing that miR-19b reduces 5-FU-induced apoptotic effects in a PPP2R5E-dependent manner (Figure S12), which reinforces the role of the miR-19b/PPP2R5E signaling axis regulating 5-FU antitumor effects in CRC cells.

## 3. Discussion

The clinical usefulness of miRs as prognostic and therapeutic biomarkers has been largely described in the last few years. However, there is a lack of data in the literature that would allow for us to completely understand their functional and therapeutic impact in the different steps of tumor progression. The clinical relevance of miR-19b deregulation in CRC and LARC as a predictive marker of both patient outcome and therapeutic response was demonstrated, but its molecular significance in these diseases remains to be fully investigated. In this regard, PPP2R5E is a regulatory subunit of the PP2A complex that is a direct target of miR-19b in NSCLC [21]. Moreover, it plays a key role determining changes in PP2A activity in several cancer models such as NSCLC and CRC [21,32].

In addition, the fact that PP2A activation status determines 5-FU response of CRC cells [34] prompted us to analyze the clinical role of the miR-19b/PPP2R5E axis in LARC due to 5-FU-based treatment is the standard neoadjuvant therapy in this disease. Thus, we identified not only a negative correlation between miR-19b and PPP2R5E in LARC patients, but also that PPP2R5E expression was predictive of pathological response to neoadjuvant treatment in this disease [29]. Moreover, miR-19b determines 5-FU resistance and predicts tumor progression in LARC [30], but the potential role of PPP2R5E in this issue was not investigated. Thus, we analyzed the role of PPP2R5E in the previously described miR-19b-mediated effects on the 5-FU response of CRC cells, and studied the functional significance of the miR-19b/PPP2R5E signaling axis in this disease. We first explored how miR-19b modulates cell viability, which was significantly enhanced after miR-19b overexpression, and reduced when this miR was silenced (Figure 1). These findings were concordant with the observed results in our previous work when we analyzed the effect of miR-19b in determining 5-FU sensitivity [30]. As expected, PPP2R5E overexpression significantly impaired cell viability whereas its silencing led to increased cell viability (Figures S1 and S2), suggesting that miR-19b-induced effects are at least partially mediated by its role as negative PPP2R5E regulator.

Our next objective was to experimentally confirm that miR-19b regulates the PPP2R5E in CRC cells, similarly as described in NSCLC. First, we used a Western blot to analyze the effect of miR-19b modulation on PPP2R5E expression, observing that miR-19b overexpression led to a reduction in PPP2R5E protein levels (Figure 2A). We next confirmed via luciferase assays the role of miR-19b as a negative post-transcriptional regulator of PPP2R5E in CRC cells (Figure 2B). This issue was concordant with the ability of this miR to modulate PP2A activity (Figure 2C and Figure S3), suggesting that miR-19b overexpression could represent a molecular alteration responsible of the PPP2R5E downregulation previously identified in CRC patients [32], which should be investigated in forthcoming studies. After demonstrating the direct miR-19b regulation of PPP2R5E, we tested the relevance of the miR-19b/PPP2R5E axis modulating cancer cell viability. Thus, we studied the effect of miR-19b modulation in CRC cells ectopically expressing PPP2R5E and observed that PPP2R5E totally inhibited the miR-19b-induced cell viability (Figure 3A). Moreover, miR-19b silencing in combination with PPP2R5E overexpression led to similar cell viability inhibition, indicating that PPP2R5E deregulation plays a key role in miR-19b-induced effects on cell viability (Figure 3B).

Furthermore, we evaluated the importance of PPP2R5E in the modulation of additional biological properties regulated by miR-19b in CRC cells, and the contribution of miR-19b/PPP2R5E axis in tumor progression through the regulation of different cellular processes. Since the PP2A pathway is involved in different cellular processes such as cell migration and colonosphere formation [35], we studied the potential contribution of the miR-19b/PPP2R5E signaling axis to the regulation of these biological functions. Both miR-19b and PPP2R5E induced significant changes in these cell properties (Figure 4 and Figure 5), reinforcing our hypothesis that the miR-19b/PPP2R5E axis plays a relevant role in CRC progression. The observed changes with miR-19b or PPP2R5E overexpression were lower in the SW480 cell line than those in HT-29 cells, which could be explained by the fact that the first showed reduced basal levels of PPP2R5E compared to the HT-29 cell line. The lack of a perfect correlation in some cases between the observed effects after the ectopic modulation of miR-19b and PPP2R5E is probably due to the miR-19b regulation of additional targets. In fact, miR-19b negatively regulates PRKAA1 and BIM in T-cell acute lymphoblastic leukemia, in addition to PPP2R5E, resulting in an overall PI3K signaling pathway activation [31]. Moreover, miR-19b overexpression led to a significant deregulation of p53 and PTEN [22,23,24], which results in an increased tumor growth and cancer progression. These miR-19b oncogenic properties can also be modulated by the activation status of additional targets and signaling pathways that are critically regulated by PP2A, such as RAS/ERK/STAT [21], reported to regulate cell growth and migration in other cancer models.

Lastly, we analyzed the importance of PPP2R5E in the miR-19b-induced changes to the 5-FU sensitivity of CRC cells reported in a recent published work [30]. Interestingly, PPP2R5E overexpression completely restored 5-FU sensitivity in miR-19b overexpressing cells, whereas PPP2R5E silencing alone or combined with miR-19b overexpression led to significantly higher cell viability rates (Figure 6). Altogether, these results confirm the role of the miR-19b/PPP2R5E axis determining response of CRC cells to 5-FU treatment. Given these results, and the fact that miR-19b predicts recurrence in LARC patients [29,30] and is significantly upregulated in CRC and colorectal advanced adenomas (CAA) patients after tumor resection [36], it would be also relevant to evaluate the potential clinical usefulness of PPP2R5E as a predictive biomarker of disease progression in LARC.

## 4. Materials and Methods

### 4.1. Cell Cultures and Transfection

The in vitro experiments were conducted using CRC cell lines SW480 (ATCC-CCL-228) and HT-29 (ATCC-HTB-38) that were authenticated by the authors (LGC Standards, Wesel, Germany). Both cell lines were grown at 37 °C in a 5% CO_2_ atmosphere using RPMI-1640 medium (Invitrogen, Carlsbad, CA, USA) supplemented with 10% fetal bovine serum and 1% antibiotics (penicillin-G and streptomycin). Transfections were carried out using Lipofectamine 2000 (Invitrogen, Carlsbad, CA, USA) and specific pre- or anti-miR-19b, and non-targeting scrambled pre- or anti-miR negative controls (Ambion, Cambridge, UK), 1 µg of PPP2R5E plasmid vector or an empty plasmidic vector as negative control, and 75 nM of PPPR5E-specific siRNAs or a silencer negative control siRNA (Dharmacon, Lafayette, CO, USA).

### 4.2. Protein Extraction

The TRIzol Reagent (Invitrogen, Carlsbad, CA, USA) was used to perform all protein extractions according to the manufacturer’s protocol. Protein concentrations were measured by BCA assays (Thermo Fischer Scientific, Waltham, MA, USA).

### 4.3. Western Blot Analysis

To perform Western blot analyses, the selected protein extracts were denatured and exposed to sodium dodecyl sulfate–polyacrylamide gel electrophoresis and Western blot. Antibodies: goat polyclonal anti-PPP2R5E (Novus Biologicals, St. Charles, MO, USA), and mouse monoclonal anti-β-actin (Sigma, St. Louis, MO, USA). Secondary antibodies were conjugated to alkaline phosphatase (Sigma, St. Louis, MO, USA) and Tropix CSPD and Tropix Nitro Block II (Applied Biosystems, Foster City, CA, USA) were used for chemoluminiscent signal detection.

### 4.4. Luciferase Assay

Prior to performing luciferase assays, SW480 cells were simultaneously transfected with pre-miR-19b and a pmiR-Glo construct including an insert with the specific miR-19b seed region (in bold) (5′ ATCTCGAGCT**TTTGCACA**TCTTCCTGAGTTGAATGTCCACGTGGTCTAGAGT 3′) located in PPP2R5E 3′UTR, or an insert with a mutated miR-19b seed region (5′ ATCTCGAGCT**TGCTAGCA**TCTTCCTGAGTTGAATGTCCACGTGGTCTAGAGT 3′). Dual-Glo Luciferase Assay System (Promega) was used to perform luciferase assays and normalized firefly luciferase activity (the ratio between firefly and renilla luciferase activities) was compared to a pmiR-Glo vector without insert as control. The bioinformatics tool Target Scan was used to predict the interaction region between miR-19b and PPP2R5E.

### 4.5. PP2A Phosphatase Activity Assay

PP2A assays were performed with 50 µg of protein extracts using a PP2A immunoprecipitation phosphatase assay kit (Millipore, Burlington, MA, USA) according to manufacturer’s instructions. Briefly, PP2A immunoprecipitation was performed adding 25 μL of Agarose-Protein A slurry and 4 μg of PP2A antibody to the protein extract in a final volume of 500 μL with TBS 1X. The mix was incubated in constant rocking during 2 h followed by 3 washes with TBS 1X and a final wash with a Ser/Thr buffer assay provided by the kit. After removing the buffer, 60 μL of phosphopeptide and 20 μL of Ser/Thr buffer were added, and the mix incubated with shaking 10 min at 30 °C. Next, 25 μL of the mix per well of a 96-well plate was used in triplicates for the Malachite Green Detection measuring absorbance at 650 nm after 15 min of incubation with 100 μL of malachite solution per well. Thus, we calculated the amount of phosphatase released (pmol) using a standard curve (0–2000 pmol).

### 4.6. Cell Viability Assay

Cell viability was evaluated by MTS assays performed in triplicate wells. The CellTiter 96 Aqueous One Solution Cell Proliferation Assay kit (Promega Corp, Madison, WI, USA) was used to perform these experiments in 96-well plates, following the manufacturer’s recommendations.

### 4.7. Transwell Migration Assay

Costar 3422 polycarbonate membranes with a pore size of 8 μm (Corning Inc., Corning, NY, USA) were used to perform transwell migration assays, seeding 1 × 10^4^ cells/mL. Final volumes of 100 μL of RPMI-1640 serum-free medium and 800 μL of supplemented RPMI-1640 were placed in the upper and bottom chambers, respectively. The cells in the lower surface of the membrane were counted after 24 h under a light microscope after being stained using crystal violet.

### 4.8. Colonosphere Formation

The generation of colonospheres from WS480 and HT-29 cells were performed in 6-well ultra-low attachment plates plating 1 × 10^4^ cells per well. The medium used included DMEM/F12 supplemented with GlutMAXTM-I (Gibco), 1% N2 (Gibco), 2% B27 (Gibco), 20 ng/mL human FGF (Sigma) and 50 ng/mL EGF (Sigma). Colonosphere formation was analyzed after 7 days of incubation quantifying the number of spheres and cells per colonosphere, which was performed by collecting the colonospheres and dissociating them in single-cell suspensions.

### 4.9. Statistical Analysis

Data represented in the different functional in vitro assays included in this study are the mean of three independent experiments ± S.D. Statistical comparisons were carried out by 2-sided *t*-test analyses. A *p* value less than 0.05 was considered statistically significant, as previously described [34].

## 5. Conclusions

We highlighted the biological relevance of miR-19b as a direct negative regulator of PPP2R5E expression in CRC cells that promotes uncontrolled cell viability, migration, and colonosphere formation, which significantly contribute to tumor progression. In addition, the miR-19b/PPP2R5E axis could have therapeutic value in this disease due to the role of miR-19b in determining 5-FU resistance through PP2A inhibition, which needs to be fully validated at the clinical level in forthcoming studies.

## Data Availability

Data sharing is not applicable for this article.

## Supplementary Materials

The supporting information can be downloaded at: https://www.mdpi.com/article/10.3390/ijms24097779/s1.
