# Supplementary material for: Deregulation of the miR-19b/PPP2R5E Signaling Axis Shows High Functional Impact in Colorectal Cancer Cells
Source: Int J Mol Sci. 2023 Apr 24;24(9):7779. doi: 10.3390/ijms24097779 (PMC10178228; doi:10.3390/ijms24097779)
Supplement: Supplementary file 1 [file ijms-24-07779-s001.zip › ijms-2290891-supplementary.pdf]

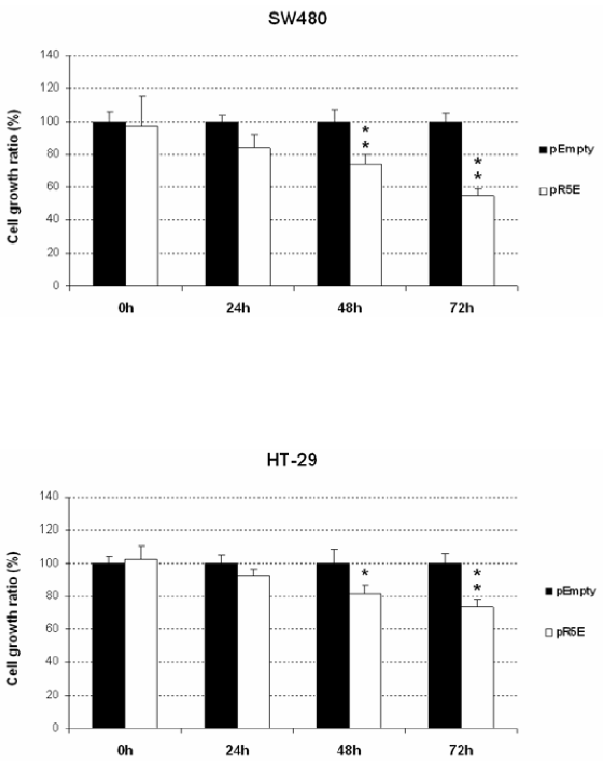

Figure S1. MTS assays showing the effects of ectopic PPP2R5E overexpression in SW480 and HT-29 cell viability; * $p < 0.05$; ** $p < 0.01$.

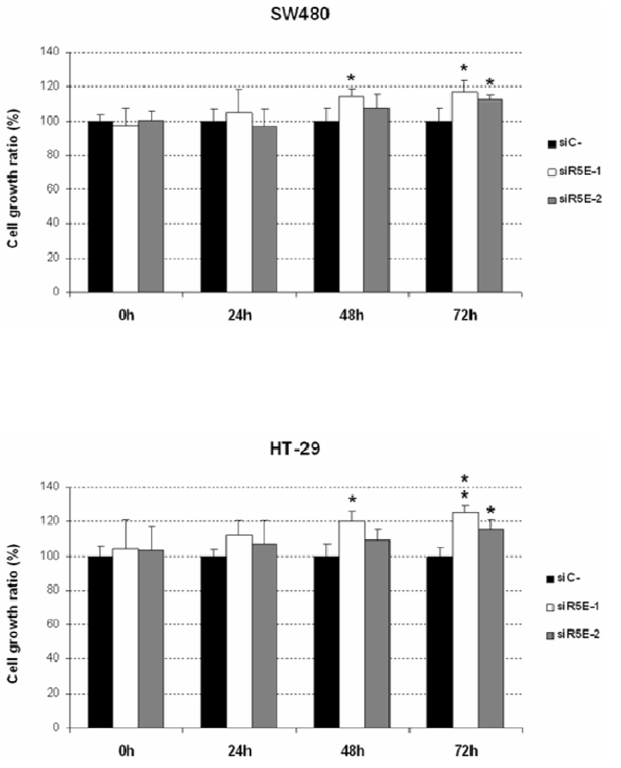

Figure S2. MTS assays showing the effects of ectopic PPP2R5E silencing in SW480 and HT-29 cell viability; * $p < 0.05$; ** $p < 0.01$.

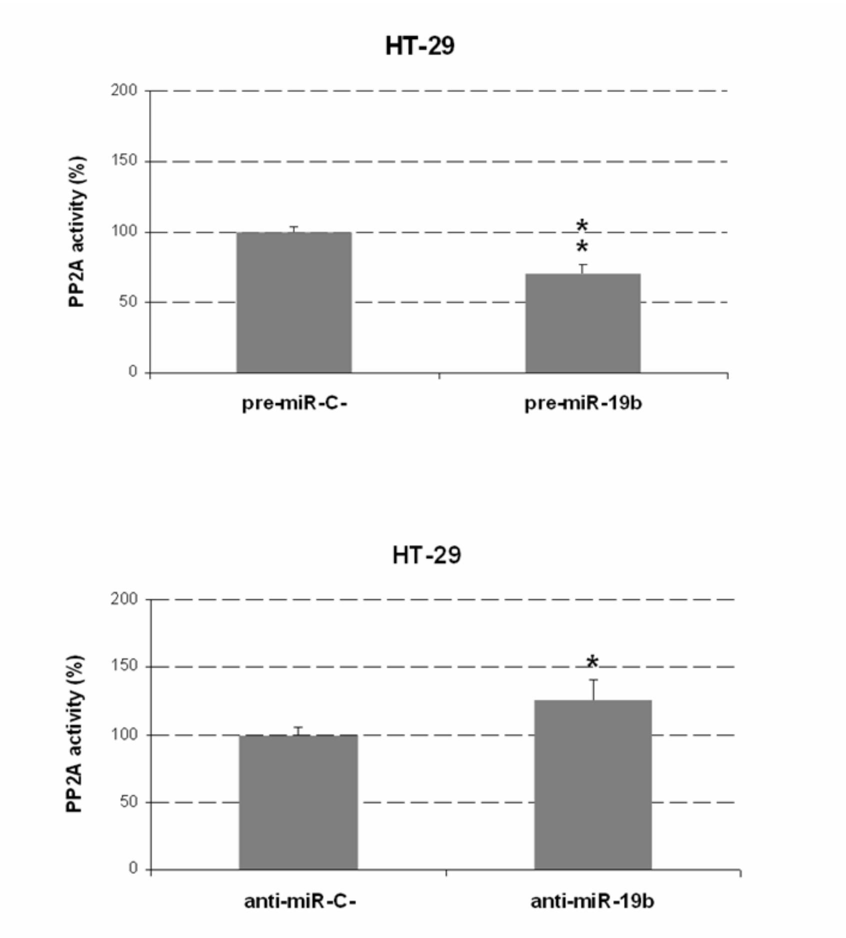

Figure S3. PP2A phosphatase activity assays in HT-29 cells after miR-19b overexpression or silencing. Graphs show the mean of three independent experiments ±SD; * $p < 0.05$; ** $p < 0.01$.

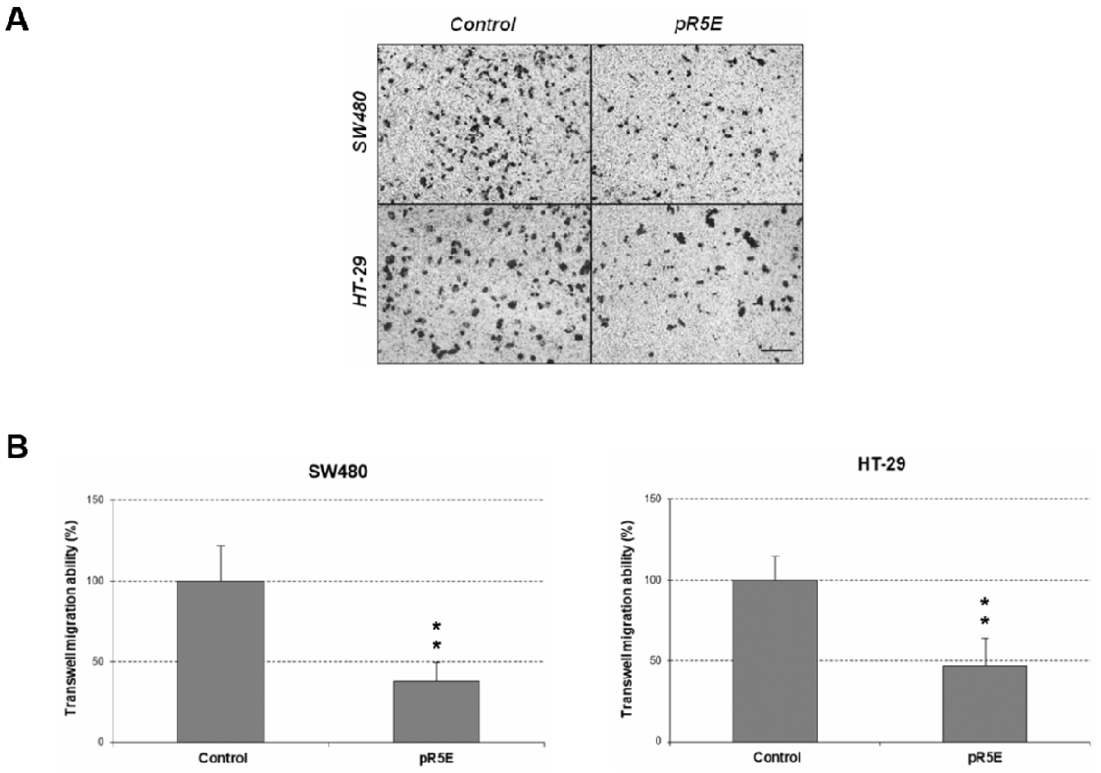

Figure S4. PPP2R5E overexpression inhibits CRC transwell migration. **(A)** Optical microscope images (amplification x100). **(B)** Changes in migration of SW480 and HT-29 cells after PPP2R5E overexpression; * $p < 0.05$; ** $p < 0.01$. Scale bar: 200 μm.

A

B

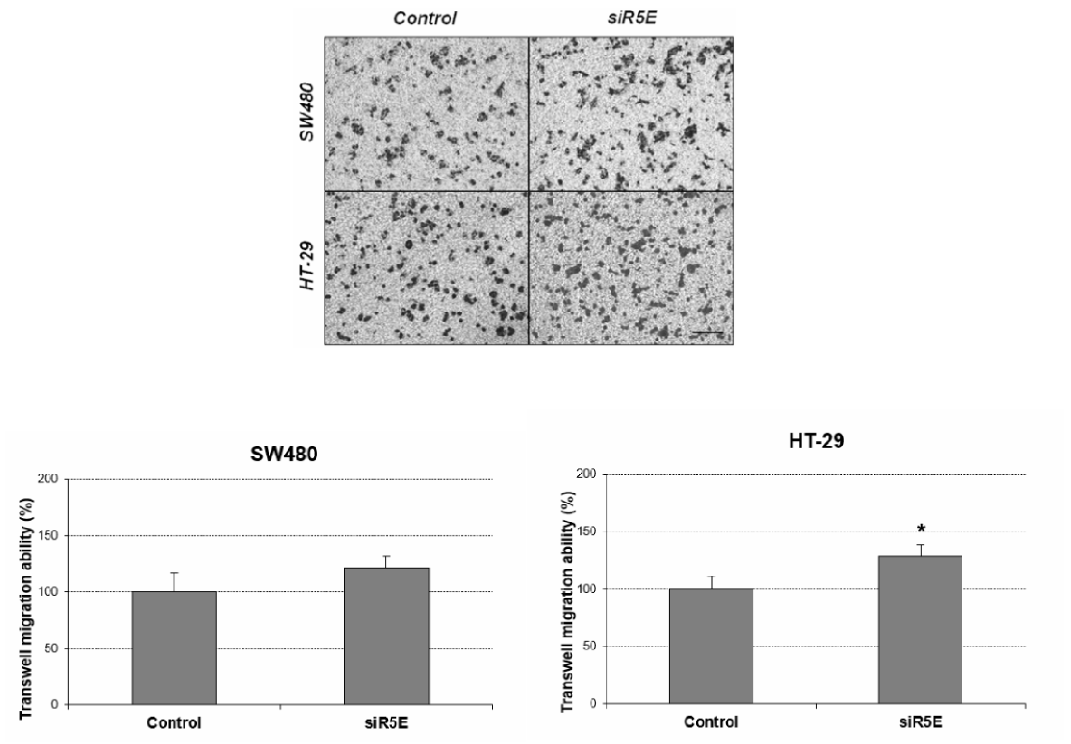

Figure S5. PPP2R5E silencing enhances CRC transwell migration. **(A)** Optical microscope images (amplification x100). **(B)** Changes in migration of SW480 and HT-29 cells after PPP2R5E silencing; * $p < 0.05$; ** $p < 0.01$. Scale bar: 200 μm.

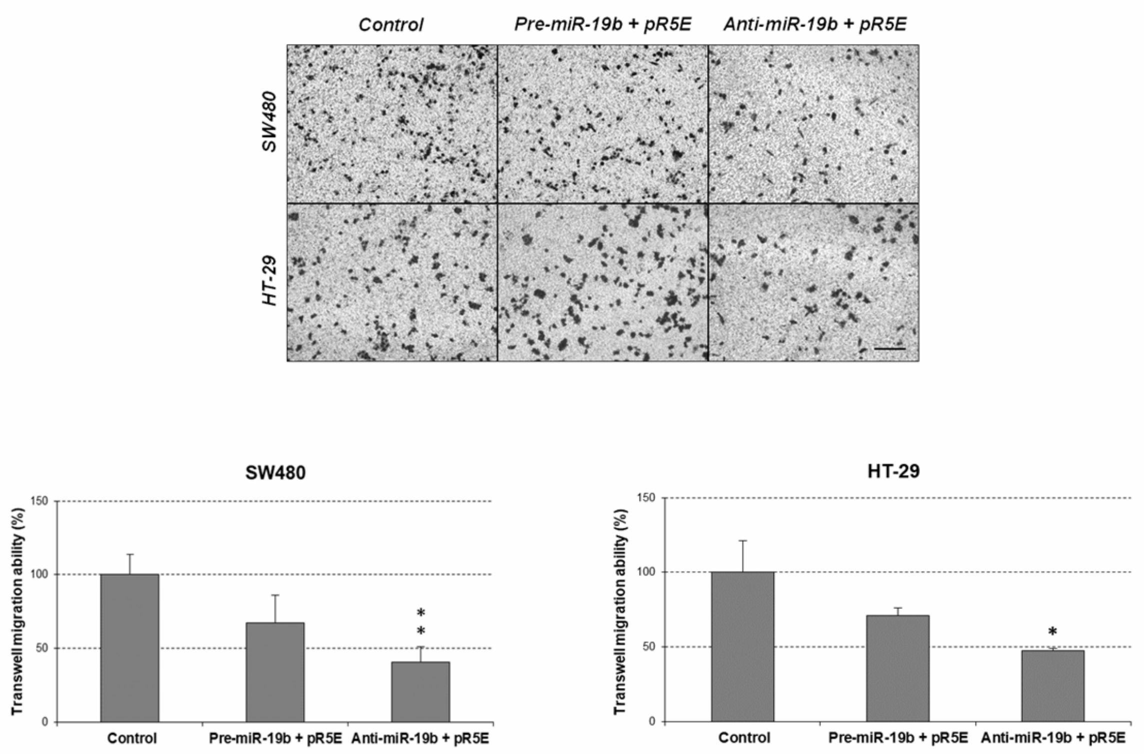

Figure S6. Effects of miR-19b modulation in cell migration of SW480 and HT-29 ectopically expressing PPP2R5E; * $p < 0.05$; ** $p < 0.01$.

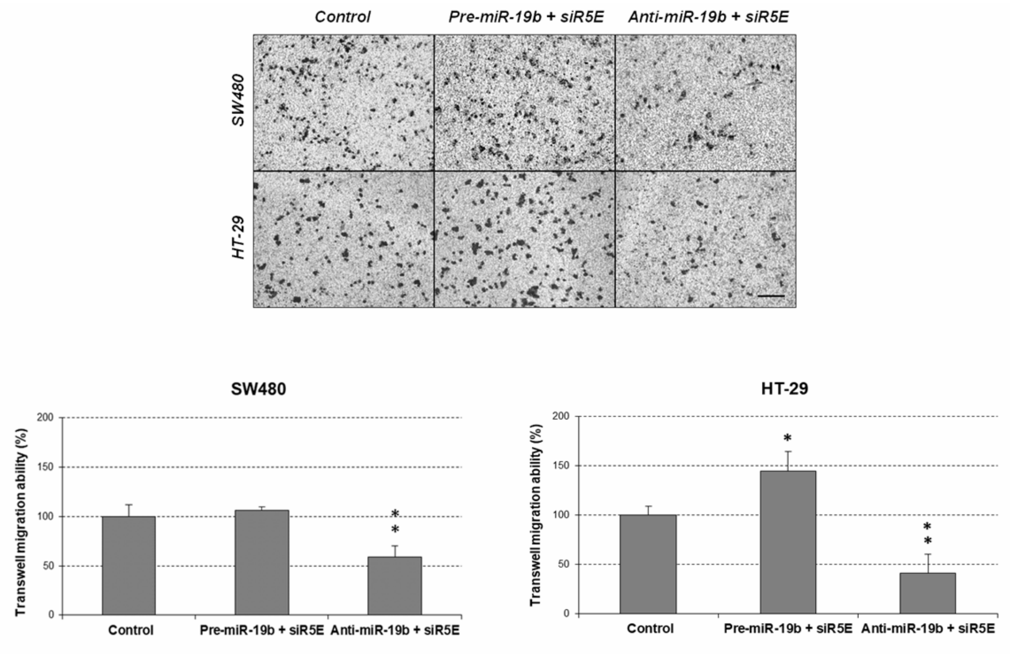

Figure S7. Effects of miR-19b modulation in cell migration of SW480 and HT-29 after PPP2R5E silencing; * $p < 0.05$; ** $p < 0.01$.

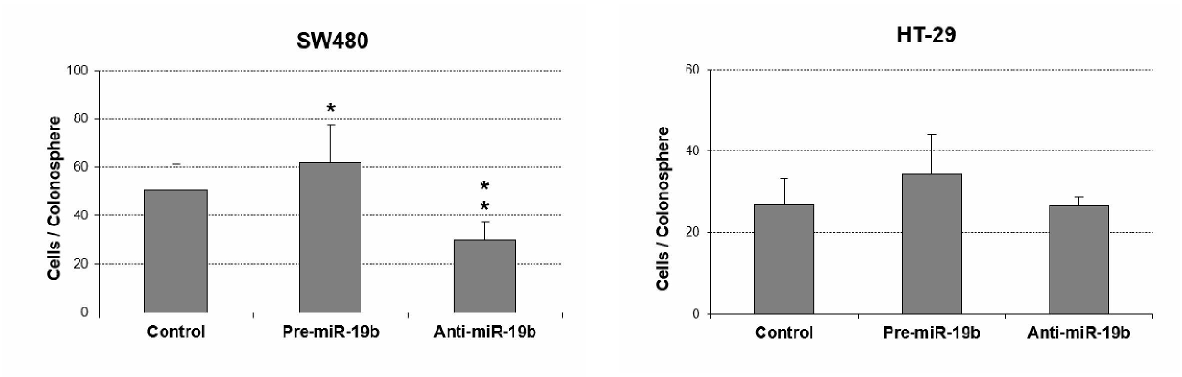

Figure S8. Effect of miR-19b in the size of the colonospheres obtained from SW480 and HT-29 cells after ectopic miR-19b modulation; * $p < 0.05$; ** $p < 0.01$.

A

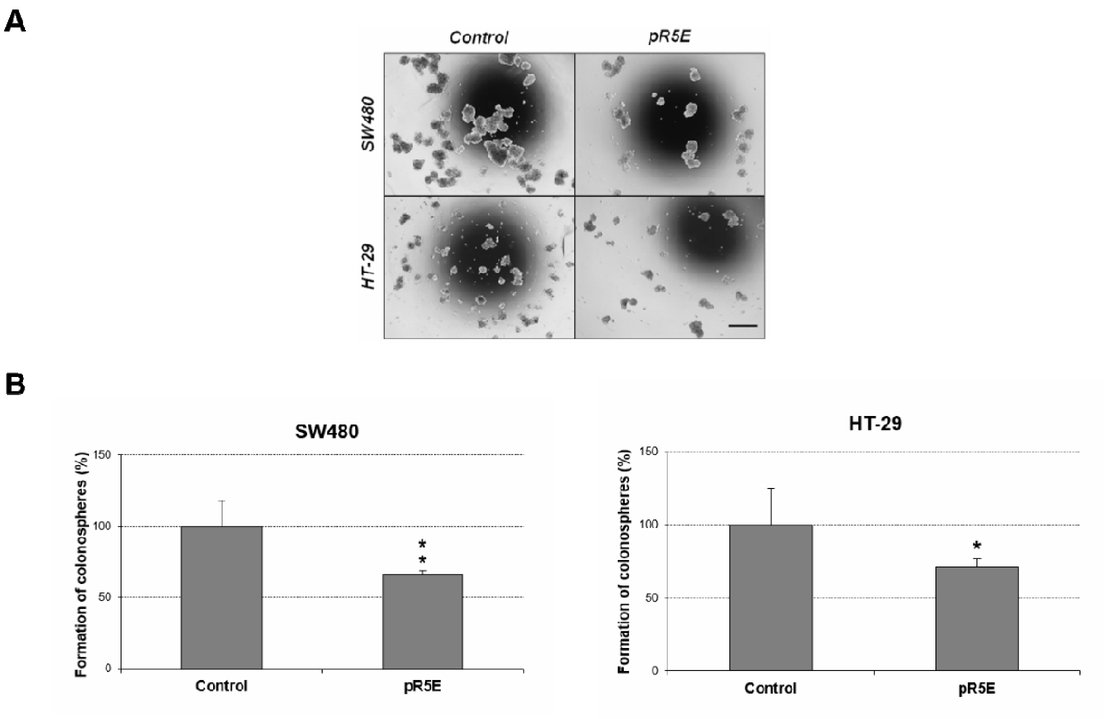

Figure S9. Effect of PPP2R5E overexpression in colonosphere formation ability. **(A)** Optical microscope images (amplification x100) showing colonospheres formed in the different conditions. **(B)** Number of colonospheres obtained from SW480 and HT-29 cells after ectopic PPP2R5E overexpression; * $p < 0.05$; ** $p < 0.01$. Scale bar: 200 µm.

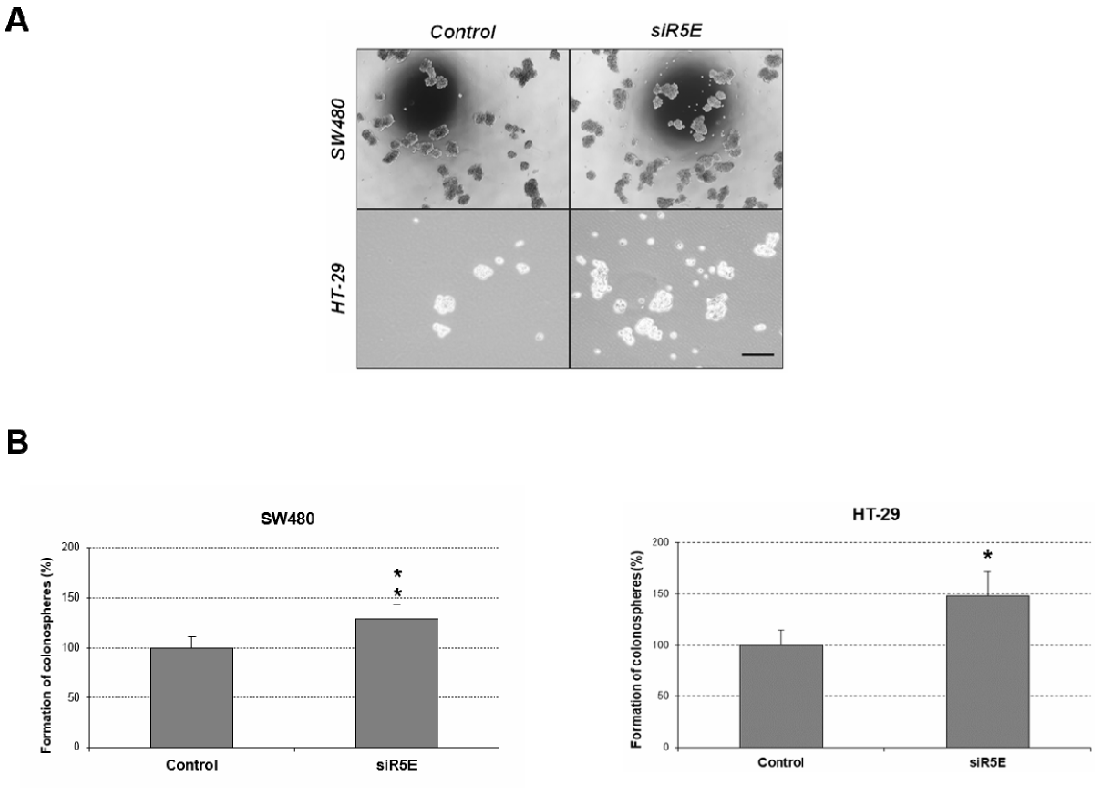

Figure S10. Effect of PPP2R5E silencing in colonosphere formation ability. **(A)** Optical microscope images (amplification x100) showing colonospheres formed in the different conditions. **(B)** Number of colonospheres obtained from SW480 and HT-29 cells after PPP2R5E silencing; * $p < 0.05$; ** $p < 0.01$. Scale bar: 200 μm.

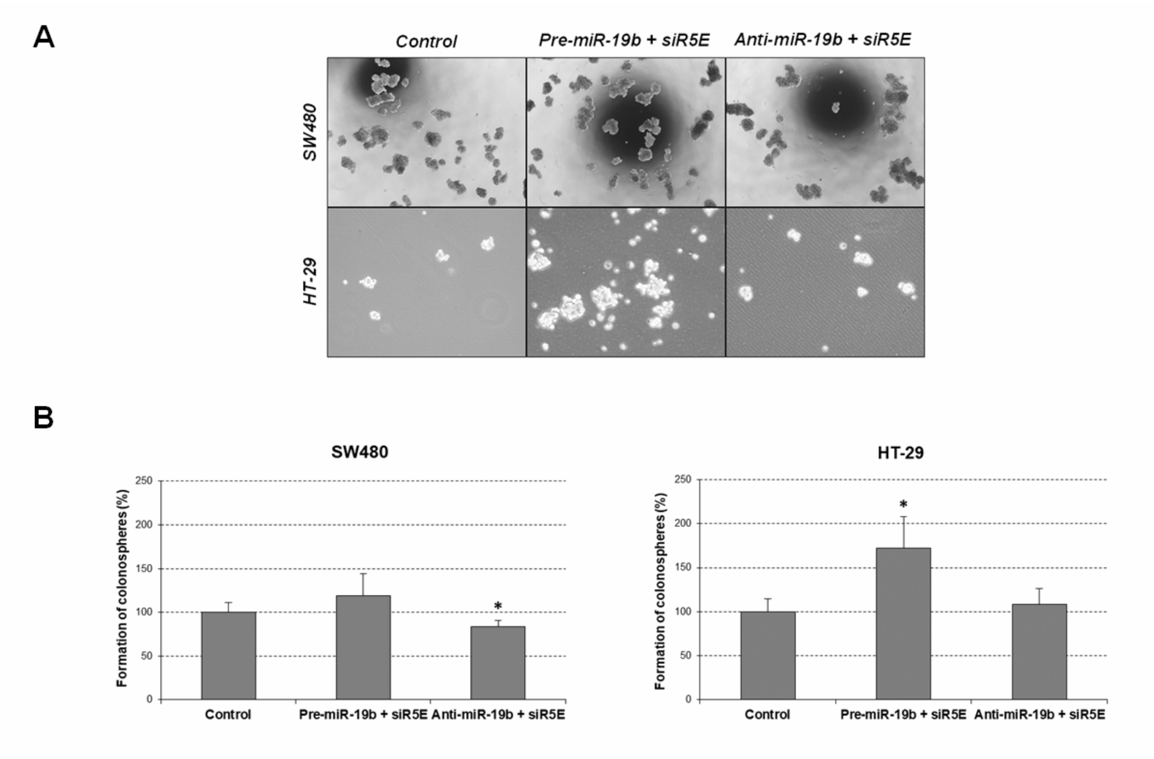

Figure S11. Regulation of colonosphere formation ability by the miR-19b/PPP2R5E axis. **(A)** Optical microscope images (amplification x100) showing colonospheres formed in the different conditions. **(B)** Number of colonospheres obtained from SW480 and HT-29 cells after transfection with pre- or anti-.miR-19b and PPP2R5E silencing; * $p < 0.05$; ** $p < 0.01$. Scale bar: 200 μm.

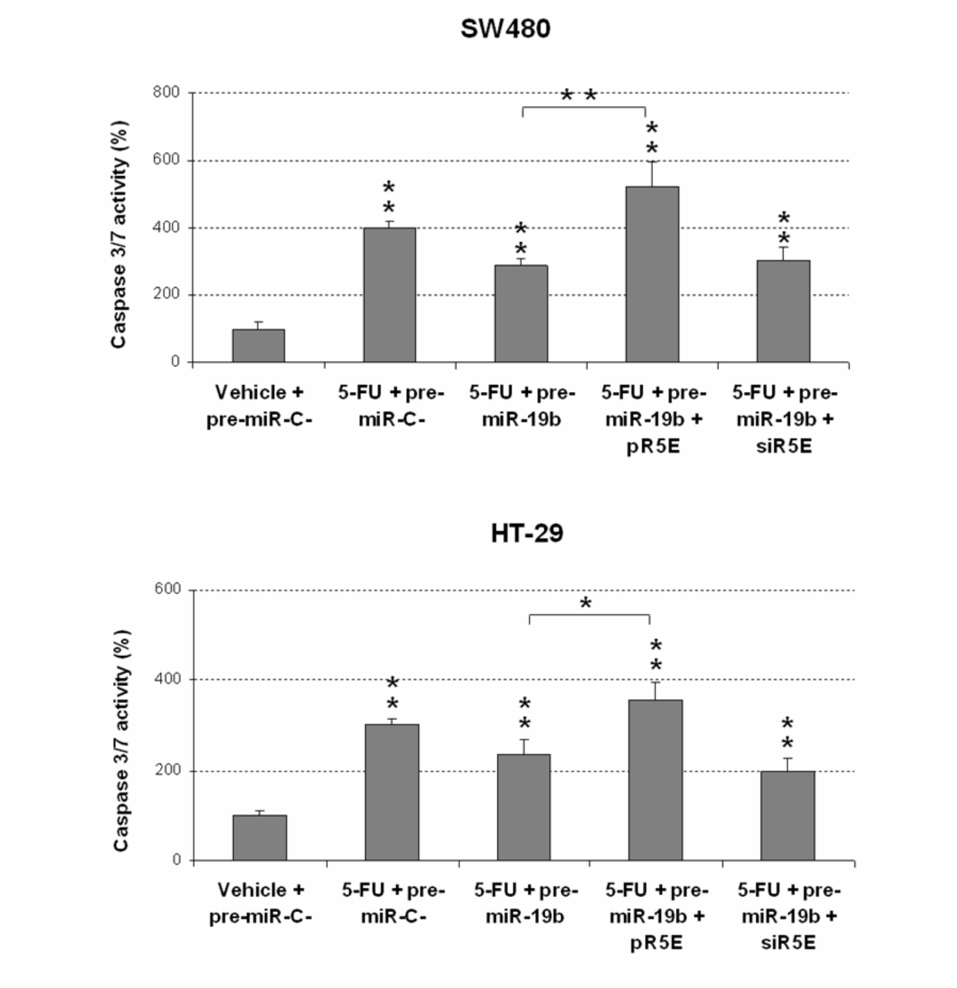

Figure S12. Caspase 3/7 assay showing the role of the miR-19b/PPP2R5E interplay in the regulation of CRC response to 5-FU treatment; * $p < 0.05$; ** $p < 0.01$.
